# Integrated Analysis of Multi-Omics Alteration, Immune Profile, and Pharmacological Landscape of Pyroptosis-Derived lncRNA Pairs in Gastric Cancer

**DOI:** 10.3389/fcell.2022.816153

**Published:** 2022-02-25

**Authors:** Chunguang Guo, Zaoqu Liu, Yin Yu, Shirui Liu, Ke Ma, Xiaoyong Ge, Zhe Xing, Taoyuan Lu, Siyuan Weng, Libo Wang, Long Liu, Zhaohui Hua, Xinwei Han, Zhen Li

**Affiliations:** ^1^ Department of Endovascular Surgery, The First Affiliated Hospital of Zhengzhou University, Zhengzhou, China; ^2^ Department of Interventional Radiology, The First Affiliated Hospital of Zhengzhou University, Zhengzhou, China; ^3^ Department of Pathophysiology, School of Basic Medical Sciences, The Academy of Medical Science, Zhengzhou University, Zhengzhou, China; ^4^ Department of Neurosurgery, The Fifth Affiliated Hospital of Zhengzhou University, Zhengzhou, China; ^5^ Department of Cerebrovascular Disease, Zhengzhou University People’s Hospital, Zhengzhou, China; ^6^ Department of Hepatobiliary and Pancreatic Surgery, The First Affiliated Hospital of Zhengzhou University, Zhengzhou, China

**Keywords:** gastric cancer, pyroptosis, lncRNA, gene pair, prognosis, mutation, immune landscape

## Abstract

**Background:** Recent evidence demonstrates that pyroptosis-derived long non-coding RNAs (lncRNAs) have profound impacts on the initiation, progression, and microenvironment of tumors. However, the roles of pyroptosis-derived lncRNAs (PDLs) in gastric cancer (GC) remain elusive.

**Methods:** We comprehensively analyzed the multi-omics data of 839 GC patients from three independent cohorts. The previous gene set enrichment analysis embedding algorithm was utilized to identify PDLs. A gene pair pipeline was developed to facilitate clinical translation *via* qualitative relative expression orders. The LASSO algorithm was used to construct and validate a pyroptosis-derived lncRNA pair prognostics signature (PLPPS). The associations between PLPPS and multi-omics alteration, immune profile, and pharmacological landscape were further investigated.

**Results:** A total of 350 PDLs and 61,075 PDL pairs in the training set were generated. Cox regression revealed 15 PDL pairs associated with overall survival, which were utilized to construct the PLPPS model *via* the LASSO algorithm. The high-risk group demonstrated adverse prognosis relative to the low-risk group. Remarkably, genomic analysis suggested that the lower tumor mutation burden and gene mutation frequency (e.g., *TTN*, *MUC16*, and *LRP1B*) were found in the high-risk group patients. The copy number variants were not significantly different between the two groups. Additionally, the high-risk group possessed lower immune cell infiltration abundance and might be resistant to a few chemotherapeutic drugs (including cisplatin, paclitaxel, and gemcitabine).

**Conclusion:** PDLs were closely implicated in the biological process and prognosis of GC, and our PLPPS model could serve as a promising tool to advance prognostic management and personalized treatment of GC patients.

## Introduction

Gastric cancer (GC) is a serious disease worldwide, ranking the fifth in incidence and fourth in mortality globally (Smyth et al., 2020; Sung et al., 2021). Recently, the treatment modalities for GC have been gradually refined and optimized, but surgical resection is still the main modality (Fuchs et al., 2017; Smyth et al., 2020; Sung et al., 2021). Disappointingly, due to the highly heterogeneous and occult course, most patients with GC are already in advanced stage when diagnosed, with 769,000 deaths globally in 2020 and the 5-y survival rate of only about 25% (Rugge et al., 2017; Eusebi et al., 2020; Yuan et al., 2020; Sung et al., 2021). Traditionally, clinical decisions of GC have been specified by the American Joint Committee on Cancer (AJCC) tumor node metastasis (TNM) staging system (Edition et al., 2017). However, sometimes the prognosis of GC patients with the same TNM stages who receive the same treatment modalities is not identical. The molecular heterogeneity between individuals might drive the clinical diversity of GC patients (Akshatha et al., 2021). Therefore, it is necessary to incorporate molecular characteristics into the prognostic evaluation of patients.

Pyroptosis is recognized as a new type of programmed cell death, which is inherently inflammatory and triggered by various pathological stimuli (including infection, cancer, and cardiovascular events) (Ayers et al., 2017). Pyroptosis is characterized by gasdermin family-mediated cell swelling, lysis, and the release of pro-inflammatory intracellular contents including interleukin (IL)-18 and adenosine triphosphate (Kovacs and Miao, 2017). Pyroptosis was initially an important pathway to antagonize cell infection, and with the deepening of research, it was found to play a linchpin role in influencing the tumor microenvironment (TME) (Ye et al., 2021). Accumulating evidence has indicated that pyroptosis impacts the prognosis of patients in multiple cancers by promoting or inhibiting the proliferation, invasion, and metastasis of tumors (Fang et al., 2020; Al Mamun et al., 2021; Lin et al., 2021). For instance, recent research revealed that pyroptosis promoted the proliferation of esophageal squamous carcinoma cells through the *PKM2-caspase-8/3-GSDME* pathway (Jiang et al., 2021). Tian et al. (2020) reported that *GSDME*-mediated pyroptosis promoted the progression of colorectal cancer by releasing *HMGB1*. Therefore, pyroptosis has profound impacts on cancers.

Long non-coding RNAs (lncRNAs), a type of non-coding RNA molecules greater than 200 nucleotides in length, play important roles in regulating the transcriptional, posttranscriptional, or epigenetic level (Al Mamun et al., 2021). Emerging evidence has indicated that the interactions between pyroptosis and lncRNAs are significant in the development of multiple complex human diseases, especially in cancers (He et al., 2020; Gao et al., 2021). Due to the important role of lncRNAs in the process of tumor cell proliferation, migration, and apoptosis, the diagnosis and prognosis of patients are affected (Liu S. J. et al., 2021). The aberrant expression of lncRNAs could regulate the biological behavior of pyroptosis-derived lncRNA (PDLs) through miRNAs and downstream pathways, further prolonging or shortening the overall survival (OS) of multiple cancers (He et al., 2020). Scientists reported a new finding that knockdown of *rp1-85f18.6* significantly promotes the pyroptosis of colorectal cancer cells by cleaving *GSDMD* (Ma et al., 2018). These findings demonstrated that identifying and exploring PDLs could promote the revelation of the complex mechanisms of cancer. Nevertheless, the clinical significance of PDLs in GC remains elusive and needs to be further explored.

Large-scale data open the gate to comprehensively explore molecular alterations and systematically develop useful biomarkers in cancers (Cai et al., 2020; Liu et al., 2021d; Liu et al., 2021f). However, cross-platforms remain the non-negligible obstacle in biomarker discovery. Thus, in this study, we proposed a novel gene pair pipeline based on relative expression orders. Using this pipeline, we successfully constructed and validated a pyroptosis-derived lncRNA pair prognostics signature (PLPPS) model with robust performance across multiple independent datasets. More importantly, we also revealed the multi-omics alteration, immune profile, and pharmacological landscape of PLPPS.

## Materials and Methods

### Data Collection and Processing

**FIGURE 1:**
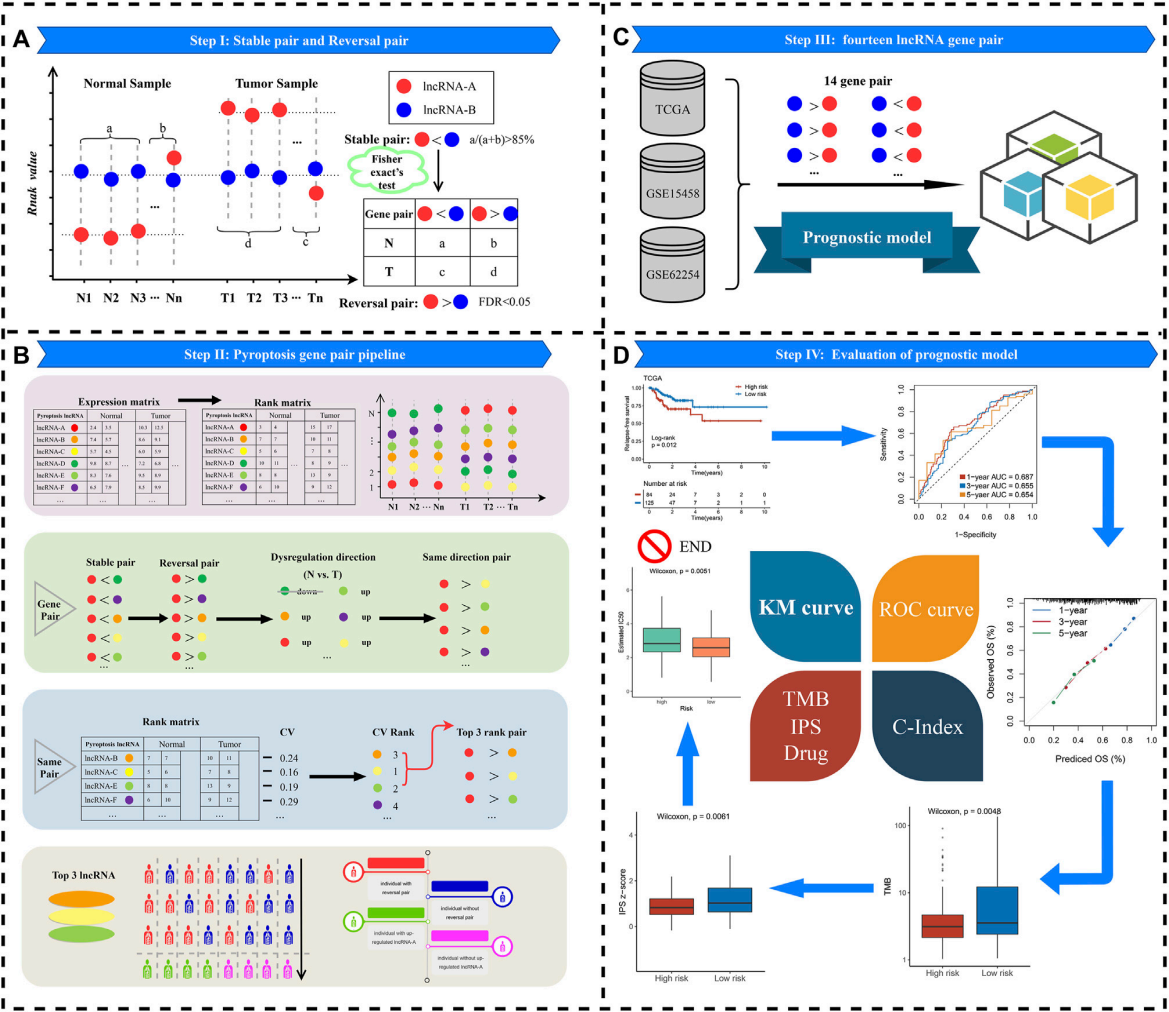
Flowchart of analysis procedure.

The flowchart of this study is shown in Figure 1. Three cohorts were enrolled from The Cancer Genome Atlas (TCGA, https://portal.gdc.cancer.gov/) and Gene Expression Omnibus (GEO, http://www.ncbi.nlm.nih.gov/geo/) datasets, including TCGA-STAD (*n* = 348), GSE62254 (*n* = 300), and GSE15458 (*n* = 191). For the TCGA-STAD cohort, “level 3” transcriptome profile and clinical data of patients were retrieved. All GEO datasets were annotated by the Affymetrix^®^ GPL570 platform, and raw transcriptome data were processed by the robust multiarray averaging (RMA) algorithm implemented in “affy” R package (Deandres-Galiana et al., 2016). In three cohorts, primary tumor tissue samples without chemoradiotherapy and cancer-adjacent normal tissues were retained. The somatic mutation and copy number variation (CNV) data were all downloaded from the TCGA portal and cBioPortal, respectively. The baseline characteristics are summarized in Supplementary Table S1.

### Pyroptosis Gene List

The pyroptosis gene list (containing a total of 51 genes) was required from the MSigDB (Liberzon et al., 2015) (version 7.4, REACTOME_PYROPTOSIS) and prior research (Shao et al., 2021; Ye et al., 2021). The gene list is illustrated in Supplementary Table S2.

### Identification of lncRNAs Associated With Pyroptosis Genes

Using our previous integrated algorithm (Liu et al., 2021c; Liu et al., 2021e), we aimed to identify the potential lncRNA regulators of pyroptosis. The summary is given as follows: 1) total mRNAs were ranked in descending order *via* their correlation with a specific lncRNA. 2) The order gene list was further entered into “fgsea” R package to perform enrichment analysis and explore whether the genes of pyroptosis signaling were enriched in the top or bottom of the ordered list. 3) The pyroptosis enrichment score (PES) was calculated for total lncRNAs. LncRNAs with significant PES were determined as pyroptosis-derived lncRNA by the permutation test framework. 4) The lncRNAs with the absolute value of PES > 0.995 and the false discovery rate (FDR) < 0.05 were determined as pyroptosis-derived lncRNAs.

### Definition of Stable and Reversal lncRNA Pairs

The lncRNA expression matrix was converted to the corresponding rank matrix (the greatest expression value was converted to the maximum rank, and the smallest expression value was converted to the minimum rank in each sample) (Figure 1A). Pairwise comparisons of all pyroptosis-derived lncRNAs were performed to determine stable pairs in normal samples and reversed pairs in tumor samples. When two lncRNAs exhibited the same rank pattern (e.g., lncRNA-A < lncRNA-B) in 85% of normal samples, these two lncRNAs were called stable pairs. Likewise, reversal pair was defined as displaying the uniform rank pattern in 85% of tumor samples and in the opposite direction to stable pairs (e.g., lncRNA-A < lncRNA-B).

### Gene Pair Pipeline

In order to screen suitable reversal pyroptosis gene pairs, we performed the following steps accordingly (Figure 1B):

Step 1: The expression profile of lncRNA was converted to the rank profile, and that will reduce batch effects during the experiment and qualitative transcriptional characterization.

Step 2: The identification of lncRNA pairs with the same direction of deregulation. For instance, lncRNA-A with lncRNA-B, lncRNA-C, lncRNA-D, lncRNA-E, and lncRNA-F constituted stable pair and reversal pair relationships in normal vs. tumor samples, respectively. Compared with normal samples, the rank of lncRNA-A in tumor samples was “up.” However, the direction of deregulation of lncRNA-D was “down.” Then, the combination of lncRNA-A with lncRNA-D will be abandoned.

Step 3: Subsequently, we calculated the coefficient of variation (CV) of partner lncRNAs for each lncRNA-A through the rank change of lncRNAs in tumor and normal tissues. Assuming that if the order of lncRNA pair is approximately invariable in normal and tumor samples, the stable and reversal relationship between lncRNA-A and partner lncRNAs may be altered because of the order change of lncRNA-A in the two groups, which facilitates judging whether lncRNA-A exists different in individual tumor samples. Based on the aforementioned reasons, we calculated the CV of patten lncRNA for lncRNA-A and ranked it in descending order. After that, the top three patten lncRNAs of the CV (lncRNA-B, lncRNA-C, and lncRNA-E) were retained and included in the following analysis (Peng et al., 2017).

Step 4:Ultimately, three lncRNA pairs (lncRNA-A > lncRNA-B, lncRNA-A > lncRNA-C, and lncRNA-A > lncRNA-E) were considered as qualified reversal pairs in every single tumor sample. For instance, more than 60% of the tumor samples had lncRNA-A > lncRNA-B, and lncRNA-A > lncRNA-B was included in the final study (the green human shape in Figure 1B).

Step 5: Relative expression ordering. For each lncRNA pair (e.g., lncRNA-A and lncRNA-B), we defined lncRNA-A > lncRNA-B as number 1. In contrast, number 0 was used to denote lncRNA-A < lncRNA-B. Passing this transformation will reduce the experimental batch effect and improve the robustness. Then, we obtained a matrix of samples based on the relative expression order of lncRNA pairs for subsequent research.

### Signature Generation

First, the stable PDL pairs were identified according to the following guideline: 1) *p*-value of univariate Cox regression less than 0.05. 2) Hazard ratio (HR) values were in the consistent direction (HR > 1 or <1) in three cohorts. Subsequently, based on the rank value of the PLPPS, we used the LASSO regression algorithm (Lockhart et al., 2014) to develop a PLPPS model to predict the OS of GC patients. To achieve this purpose, the calculation process was performed in “glmnet” R package and the lambda value was chosen when the partial likelihood deviance reached the minimum value by the 10-fold cross-validations (Friedman et al., 2010). Finally, the lncRNA pairs with non-zero coefficients were selected to establish the final prognosis model. Patients were divided into high- and low-risk groups according to the optimal cutoff point. The predictive ability of the PLPPS model was validated in GSE62254 and GSE15458.

### Copy Number Variation Analysis

Afterward, we performed a comprehensive analysis of copy number deletion and amplification of two groups. The top 10 genes in the frequency of homozygously deleted (HOMDEL) and high-level gene amplification (AMP) events were depicted based on the CNV data from cBioPortal.

### Immunogenicity Characterization Analysis

In order to decipher the immunogenicity of the two groups, tumor mutation burden (TMB) and immunophenoscore (IPS) of patients were explored. Using the “*tmb*” function in “maftool” R package, we calculated the difference of TMB between the two group patients. After that, the IPS was used to assess the immune state of each patient. IPS is an assessment protocol that quantifies tumor immunogenicity through multiple immune markers (Charoentong et al., 2017). This score has a positive correlation with immunogenicity.

### Delineate the Mutation Landscape

We compared molecular mutation differences between high- and low-risk groups. The calculation of genes with the top 20 mutation frequencies and the depiction of mutation waterfall plots were performed by “maftools” R package.

### Gene Set Enrichment Analysis

The differential genes between the high- and low-risk groups were identified by “limma” package and sequenced by the log2 (fold change) value. Subsequently, gene set enrichment analysis (GSEA) was used to decipher the underlying biological mechanisms of the GC sample using GO and KEGG terms (Molecular Signatures Database, version: c5.go.v7.4.symbols.gmt and c2.cp.kegg.v7.4.symbols.gmt).

### Immune Landscape Description

Tumor infiltration by immune and stromal cells was evaluated by the MCPcounter algorithm (Becht et al., 2016), implemented in “MCPcounter” R package. Additionally, to analyze the response to immune checkpoint blockade, the two groups’ samples were scored by T-cell inflammatory signature (TIS). TIS was constituted by 18 inflammatory genes associated with adaptive immune resistance, antigen presentation, and chemokine expression (Ayers et al., 2017).

### Chemotherapy Drug Response

In order to assess the clinical significance of the PLPPS model, we analyzed the response [evaluated by half maximal inhibitory concentration (IC50)] to chemotherapy drugs between the high- and low-risk groups. This process was implemented in “pRRophetic” R package.

### Statistical Analysis

All data processing, statistical analysis, and plotting were conducted in R 4.0.3 software. Continuous variables and categorical variables were compared between two groups using the Wilcoxon rank-sum test and Fisher’s exact test, respectively. The optimal cutoff point was used to classify patients into high- and low-risk groups. The Kaplan–Meier method and the log-rank test were utilized to estimate the different OS and relapse-free survival (RFS) between two groups. The Benjamin–Hochberg method was used to further calculate the FDR. For every analysis, statistical significance was considered at *p* < 0.05.

## Results

### Identification of PDLs and Reversal lncRNA Pairs

We identified PDLs through the integration lncRNA pipeline. Based on the lncRNA and mRNA expression datasets, the pipeline could systematically and accurately trace lncRNA molecules which are potentially linked to pyroptosis genes. Thus, a total of 350 PDLs were selected for the following research. Subsequently, PDL expression profiles were transformed to rank profiles in each GC sample. A total of 61,075 lncRNA pairs were obtained from 350 PDLs. According to the pipeline in Figure 1B, we identified 5,046, 7,753, and 2,373 reversal lncRNA pairs from TCGA-STAD, GSE62254, and GSE15458, respectively. The three lists of lncRNA pairs had 430 overlaps. Afterward, to evaluate the performance of reversal pairs between different platforms, the common 430 lncRNA pairs between sequencing and microarray datasets underwent all analyses.

### Construction and Validation of the PLPPS Model

**FIGURE 2:**
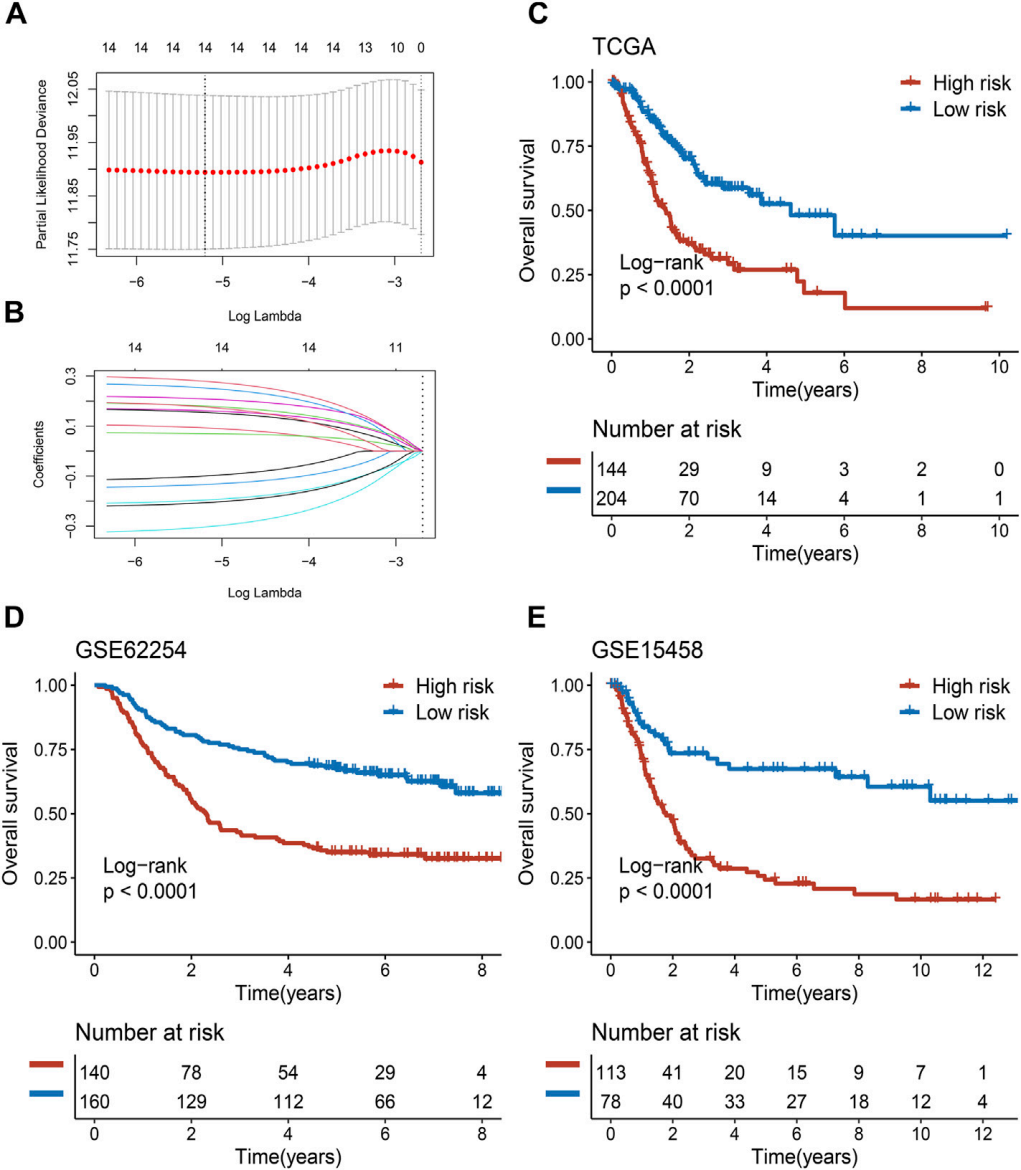
Development and validation of the PPSM model in clinical samples. **(A,B)** Least absolute shrinkage and selection operator (LASSO) logistic regression algorithm to screen of gene pairs associated with prognostic. **(C–E)** Kaplan–Meier curves of OS according to the high- and low-risk groups in TCGA **(C)**, GSE62254 **(D)**, and GSE15458 **(E)** cohorts.

**FIGURE 3:**
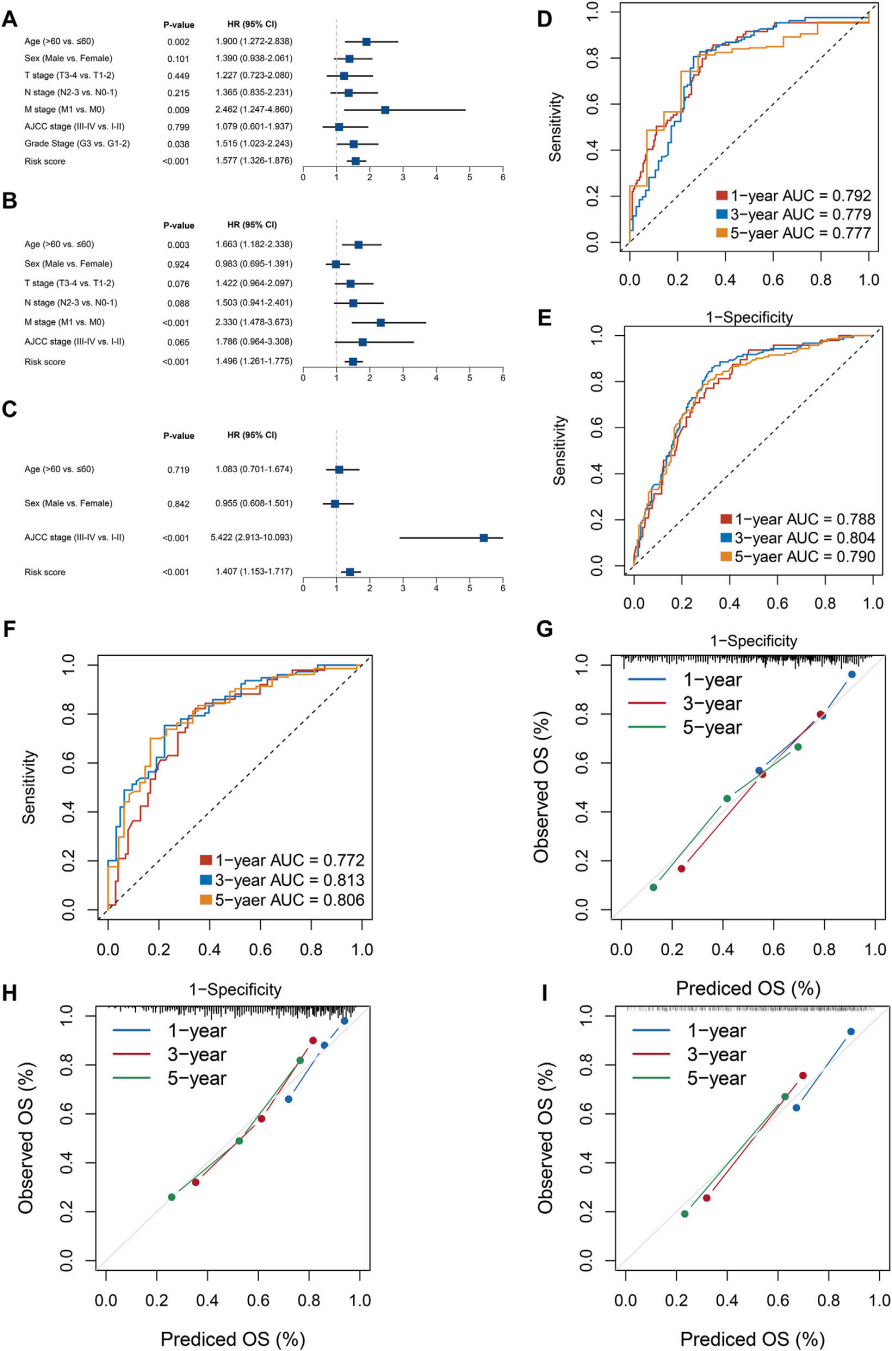
Evaluation of the PPSM model effectiveness in three cohorts. **(A–C)** Multivariate COX regression analysis of the risk score in the three cohorts: TCGA **(A)**, GSE62254 **(B)**, and GSE15458 **(C)**. **(D–F)** ROC analysis for the three cohorts: TCGA **(D)**, GSE62254 **(E)**, and GSE15458 **(F)**. **(G–I)** Calibration plots were used to compare the actual probabilities and the predicted probabilities of OS in the three cohorts: TCGA **(G)**, GSE62254 **(H)**, and GSE15458 **(I)**.

Among the 430 lncRNA pairs, we identified 15 PDLs correlated with OS by univariate Cox analysis in the three cohorts. Based on the rank profile of these lncRNA pairs in TCGA-STAD, we fitted a LASSO Cox regression model and identified 14 PDLs that were highly predictive of OS (Figures 2A,B). The specific calculation formula of risk score is given in Table 1. All patients were assigned to the high- and low-risk groups according to the optimal cutoff point. Compared with the low-risk group, patients in the high-risk group had dismal OS in all cohorts (log-rank test, all *p* < 0.05; Figures 2C–E). In addition, PLPPS remained an independent prognostic factor for GC patients after controlling the available clinical characteristic in the three cohorts (all *p* < 0.05; Figures 3A–C). Of note, patients in the high-risk group also had significantly worse RFS (Supplementary Figure S1). Multivariate Cox regression analysis displayed that PLPPS was still an independent prognostic factor for RFS (*p* < 0.05; Supplementary Figure S2).

**TABLE 1:** List of gene pairs and corresponding coefficient.

Signature	Gene A	Gene B	Coefficient
Pair 1	LINC00607	C5orf17	0.2793
Pair 2	TUSC8	PITRM1-AS1	0.2525
Pair 3	TRPM2-AS	RP11-579D7.4	0.2088
Pair 4	AC074286.1	AC058791.1	0.1802
Pair 5	MMP25-AS1	RP11-876N24.5	0.1766
Pair 6	LINC01094	RP4-680D5.8	0.1616
Pair 7	AC013275.2	RP11-567C2.1	0.1548
Pair 8	MLLT4-AS1	RP11-21L23.2	0.0930
Pair 9	LINC00607	RP11-109E24.1	0.0700
Pair 10	C10orf91	TRPM2-AS	−0.0999
Pair 11	LINC01588	RP11-73K9.2	−0.1337
Pair 12	RP3-522D1.1	LINC01094	−0.1959
Pair 13	RP11-61A14.1	RP11-416I2.1	−0.2067
Pair 14	CTD-2377D24.6	LINC01169	−0.3038

### Assessment of the PLPPS Model

In our research, we assessed this model from two perspectives: discrimination and calibration. To achieve this purpose, ROC curves and calibration plots were utilized to evaluate the PLPPS model. As shown in Figures 3D–F, the AUCs for predicting OS at 1, 3, and 5 years were 0.792, 0.779, and 0.777 in TCGA-STAD; 0.788, 0.804, and 0.790 in GSE62254; and 0.772, 0.813, and 0.806 in GSE15458, respectively. To evaluate the accuracy of the model’s prediction results, we calculated the concordance index of the PLPPS model, which were 0.737 (95% confidence interval (CI): 0.694–0.781), 0.722 (95%CI: 0.684–0.760), and 0.723 (95%CI: 0.676–0.769) in the three cohorts, respectively (Supplementary Figure S3). In addition, calibration plots displayed excellent calibration of the model that indicated the predicted probability of OS at 1, 3, and 5 years was accurate (Figures 3G–I).

### Copy Number Variation Analysis

**FIGURE 4:**
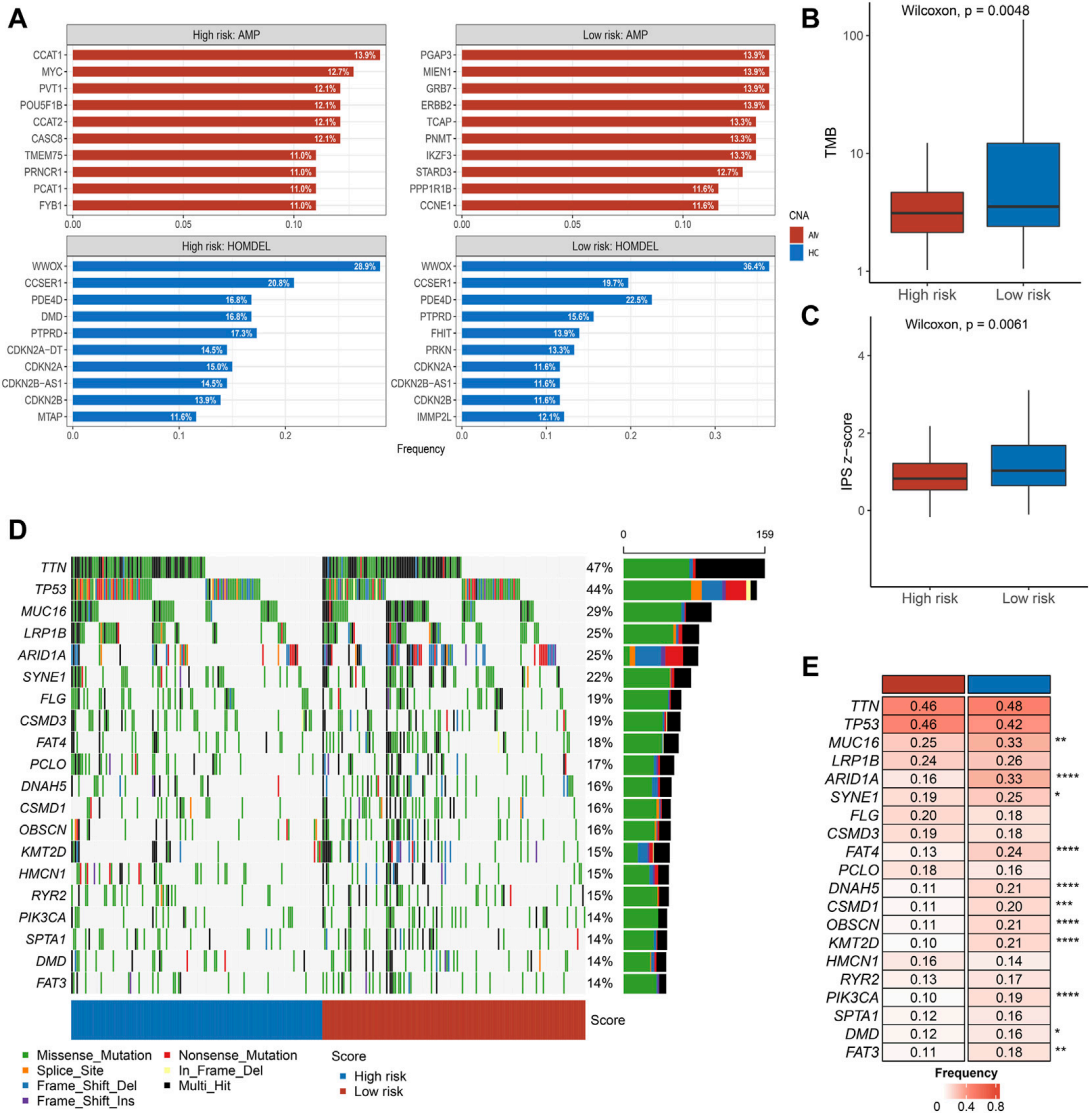
Multi-omics alteration of the PLPPS model **(A–C)**. The differences in CNV **(A)**, TMB **(B)**, and IPS **(C)** between the two groups. **(D)**. Waterfall plot of 20 frequently segments in the two subtypes **(E)**. Comparison of mutation frequency of top 20 segments.

Subsequently, we performed a comprehensive analysis of CNV in the two groups of patients. As presented in Figure 4A, we calculated the top ten genes with deletion and amplification in the two groups, respectively. We found that there was no significant difference in copy number deletion between the groups, and a high similarity was presented in the top ten genes of deletion between the two groups of patients, such as *WWOX*, *CCSER1*, *PDE4D*, and *PTPRD*. Notably, compared with low-risk group patients, patients in the high-risk group had scarcer AMP, such as *PGAP3*, *MIEN1*, *GRB7*, and *ERBB2*. The significant difference in AMP could be a potential factor for dismal prognosis in high-risk group patients.

### Immunogenicity Characterization

Tumors with higher TMB are more likely to express recognizable neoantigens that enhance the immune response. Similarly, IPS is a scoring scheme to assess the immunogenicity of tumor samples. Based on the earlier reasons, we further assessed the difference in TMB and IPS scores between the high- and low-risk groups. Notably, compared with the low-risk group, patients in the high-risk group showed inferior TMB and IPS scores (Figures 4B,C).

### Landscape of Gene Mutations in STAD

The waterfall plot delineated the top 20 high-frequency genes in the high- and low-risk groups (Figure 4D). Subsequently, we compared the frequency difference of the top 20 genes in the two groups. Overall, patients in the high-risk group displayed lower mutation frequency, especially in *MUC16*, *ARID1A*, *FAT4*, *DNAH5*, etc. (Figure 4E).

### Gene Set Enrichment Analysis

**FIGURE 5:**
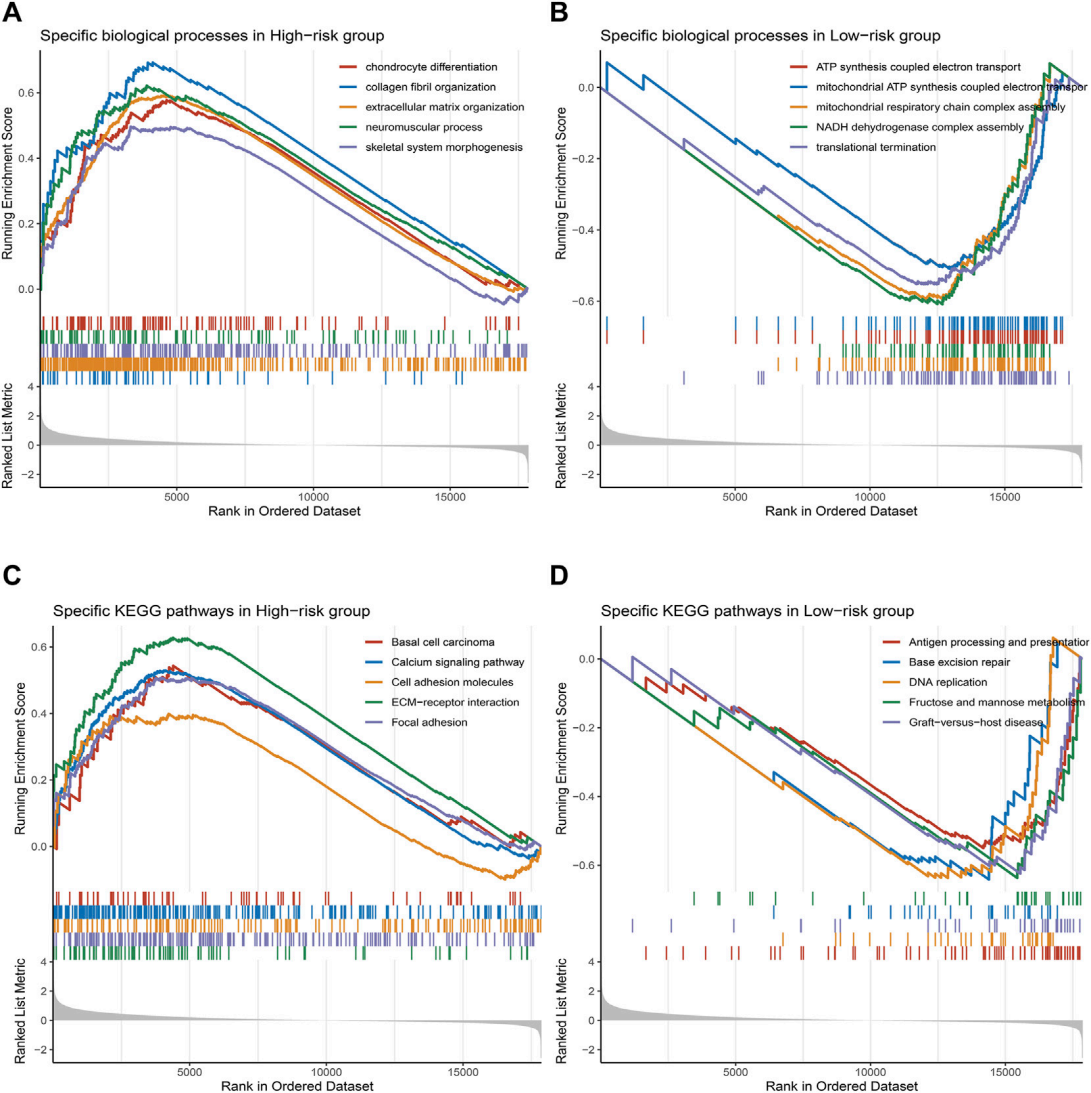
GSEA and prediction of chemotherapy response. **(A–D)**. The top five GO terms **(A,B)** and KEGG pathways **(C,D)** in the two subtypes.

GSEA revealed significant GO biological processes (Figures 5A,B) and KEGG pathways (Figures 5C,D) between the two risk groups. For biological processes, the high-risk group mainly focused on supporting and development of cellular structures, such as “chondrocyte differentiation,” “collagen fibril organization,” and “extracellular matrix organization” (Figure 5A); the low-risk group mainly acted on the generation and transmission of energy, such as “ATP synthesis coupled electron transport” and “mitochondrial respiratory chain complex assembly” (Figure 5B). For KEGG pathways, the high-risk group mainly focused on the intercellular adhesion, such as “cell adhesion molecules” and “focal adhesion” (Figure 5C); the low-risk group mainly acted on the inflammatory and immune infiltration-related functions, such as “B-cell activation” and “interleukin-6 production” (Figure 5D). Together, compared with the low–high group, high-risk group patients were not satisfactory in the enrichment of immune.

### Immune Infiltration Analysis and Prediction of Chemotherapy Response

**FIGURE 6:**
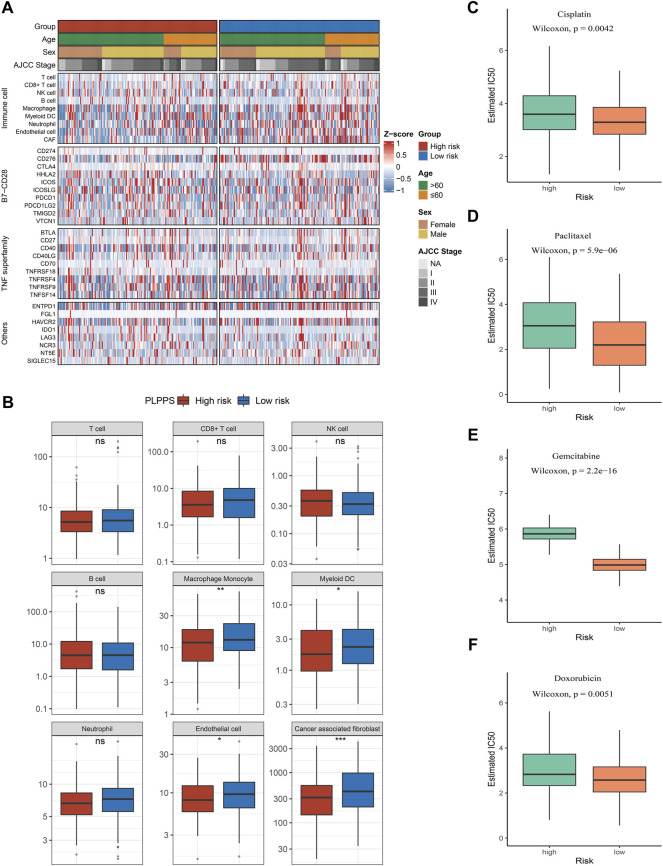
Immune infiltration analysis and prediction of chemotherapy response in the two groups. **(A)** Heatmap for immune analysis among the two subtypes. **(B)** Boxplots of nine immune cell enrichment levels **(C–F)**. Sensitivity of cisplatin **(C)**, paclitaxel **(D)**, gemcitabine **(E)**, and doxorubicin **(F)** in the two subtypes.

To further explore the infiltration abundance of immune cells between the high- and low-risk groups, the MCPcounter algorithm was utilized. We found higher overall infiltration in the low-risk group than in the high-risk group (Figure 6A); especially, the high-risk group possessed inferior levels of macrophage monocytes, myeloid dendritic cells, endothelial cells, and cancer-associated fibroblasts compared with the low-risk group (Figure 6B). Ultimately, we have chosen the cisplatin (CDDP, platinum chemotherapy drug), paclitaxel (PTX, taxus chemotherapy drug), gemcitabine (GEM, pyrimidines drug), and doxorubicin (ADM, anthracycline chemotherapy drug) to compare the responses of patients in the two subtypes to chemotherapy drugs. Remarkably, compared with the high-risk group, IC50 of the four drugs was lower in the low-risk group (Figures 6C–F).

## Discussion

Numerous reports indicated that pyroptosis exerts a dual role in tumor progression and treatment (He et al., 2020; Loveless et al., 2021). The double-edged sword roles were manifested in the phenomenon that pyroptosis promotes the evolution of normal cells into tumor cells by releasing inflammatory factors and further lead to programmed cell death of tumor cells through the infectious pathway to inhibit tumor progression (Kovacs and Miao, 2017; Ruan et al., 2020; Al Mamun et al., 2021). However, most research studies related to pyroptosis in tumors have focused on the encoded protein level, while non-coding RNAs that play an important role in apoptosis vs. cycle regulation processes have been neglected (Song et al., 2021; Ye et al., 2021; Zhao et al., 2021). Likewise, PDLs in GC also awaits further investigation. In order to eliminate the errors caused by sequencing platforms and different sample cohorts and to further improve the rigor of our research, we applied a new algorithm (Qi et al., 2016; Peng et al., 2017; Chen et al., 2018; Zhao et al., 2021) based on the gene expression ranks to reveal for the first time the impact of PDLs on patient prognosis in GC and delineate the different immune and mutational landscapes of the high- and low-risk groups.

Prognostic prediction is crucial for further interventions. In our study, we first identified 14 PDLs and elucidated their prognostic value in three independent GC cohorts. The prognostic pyroptosis signature can successfully categorize patients into subtypes with different survival outcomes. Subsequently, we found that genomic differences in patients with subgroups were abundant. As is well known, the TMB is associated with tumor immunity, and low TMB is a poor prognostic factor in GC (Roh et al., 2017; Ma et al., 2021). This is consistent with the results of the correlation analysis in our study, where patients in the high-risk group had lower TMB. Additionally, patients in the high-risk group had a higher frequency of *TP53* mutation, which is perhaps the reason for the shorter OS and RFS of patients in the high-risk group (Wang et al., 2021).

To further reveal the intrinsic biological mechanisms underlying the differences in prognostic among patients with subtypes, we performed GSEA for patients in the high- and low-risk groups. We found that inflammation and immune response-related pathways were particularly scarce in high-risk patients. Interestingly, as reported by Rao et al. (2021), “immune desert” tumors indicate adverse clinical outcomes. In addition, numerous studies indicate that high immune cell infiltration in GC patients was associated with a better long-term prognosis (Liu et al., 2020; Akshatha et al., 2021; Li et al., 2021). Notably, our investigation further suggested that differences between the abundance of immune cell infiltrates and the expression levels of immune checkpoints among the two groups. On comparing these levels in the high- and low-risk groups, we found that the levels of macrophage monocytes, myeloid dendritic cells, T cells, and CD8+ T cells were deserted in the high-risk group. On the other hand, we found that IPS scores representing immune checkpoints were similarly inferior in the high-risk patients. Our findings were consistent with the previous studies (Liu et al., 2020; Li et al., 2021; Rao et al., 2021), which indicate that PLPPS could guide the prognosis of GC patients. Different from the previous study focusing on the prognosis of GC, we further predicted sensitivity to chemotherapeutic agents in high- and low-risk group patients. The first-line chemotherapy drugs for GC such as cisplatin, paclitaxel, and gemcitabine (Joshi and Badgwell, 2021) showed higher sensitivity in the low-risk group. This further demonstrates that PLPPS has the potential to significantly contribute to identifying high-risk patients in the clinics.

Following the development of high-throughput genetic sequencing technology, we are faced with a large amount of biological data. With the combination of multi-omics with advanced algorithms, researchers can explore the molecular characteristics of GC patients in detail. This has led to the emergence of abundant prognostic and predictive gene expression signatures (Liu et al., 2021b; Wang et al., 2021; Zhang et al., 2021; Zhao et al., 2021). Nevertheless, these signatures are difficult to implement clinically due to the difficulty in unifying sequencing platforms and samples. To address these issues, we used the gene pair algorithm (Qi et al., 2016; Peng et al., 2017). As the gene pair was calculated based on the gene expression ranking of tumor samples, they could be personalized for various platform data without normalization. This indicates that our signature built from 14 PDLs more clinically applicable.

Our work is the first to comprehensively evaluate the prognostic characteristics of GC patients by PDLs. Our research has the following advantages: 1) we identified PDLS by a novel algorithm: lncRNA pipeline. 2) The gene expression order was used, instead of gene expression quantity, which eliminates the influence of different platforms and cohorts on the prognostic model and improves its applicability. 3) The PLPPS model was validated in multiple independent cohorts, ROC curves, concordance index, and calibration plots all displaying that the model has high accuracy. 4) This study comprehensively depicted the immune and mutational landscape of patients in two GC subgroups and predicted their differential sensitivity to multiple chemotherapeutic agents that demonstrate the accuracy and clinical applicability of the model from multiple aspects. Although further biological validation is necessary, we believe that comprehensive analysis based on the multicenter and the larger sample can compensate for the shortcoming.

## Conclusion

In conclusion, we proposed a gene pair pipeline that could ignore the platform batch. Based on this pipeline, the PLPPS consisting of 14 PDL gene pairs was constructed and validated across multiple independent cohorts. This signature was verified to serve as a promising and reliable prognostic tool in GC. In addition, the multi-omics alteration, immune profile, and pharmacological landscape of PLPPS were further revealed, which could provide new insights for individualized treatment and management of GC patients.

## Data Availability

The datasets presented in this study can be found in online repositories. The names of the repository/repositories and accession number(s) can be found in the article/Supplementary Material.
