# Supplementary material for: Integrated Analysis of Multi-Omics Alteration, Immune Profile, and Pharmacological Landscape of Pyroptosis-Derived lncRNA Pairs in Gastric Cancer
Source: Front Cell Dev Biol. 2022 Feb 25;10:816153. doi: 10.3389/fcell.2022.816153 (PMC8916586; doi:10.3389/fcell.2022.816153)
Supplement: Supplementary file 2 [file DataSheet1.docx]

**Supplement Figure**

- **Figure S1**
- **Figure S2**
- **Figure S3**


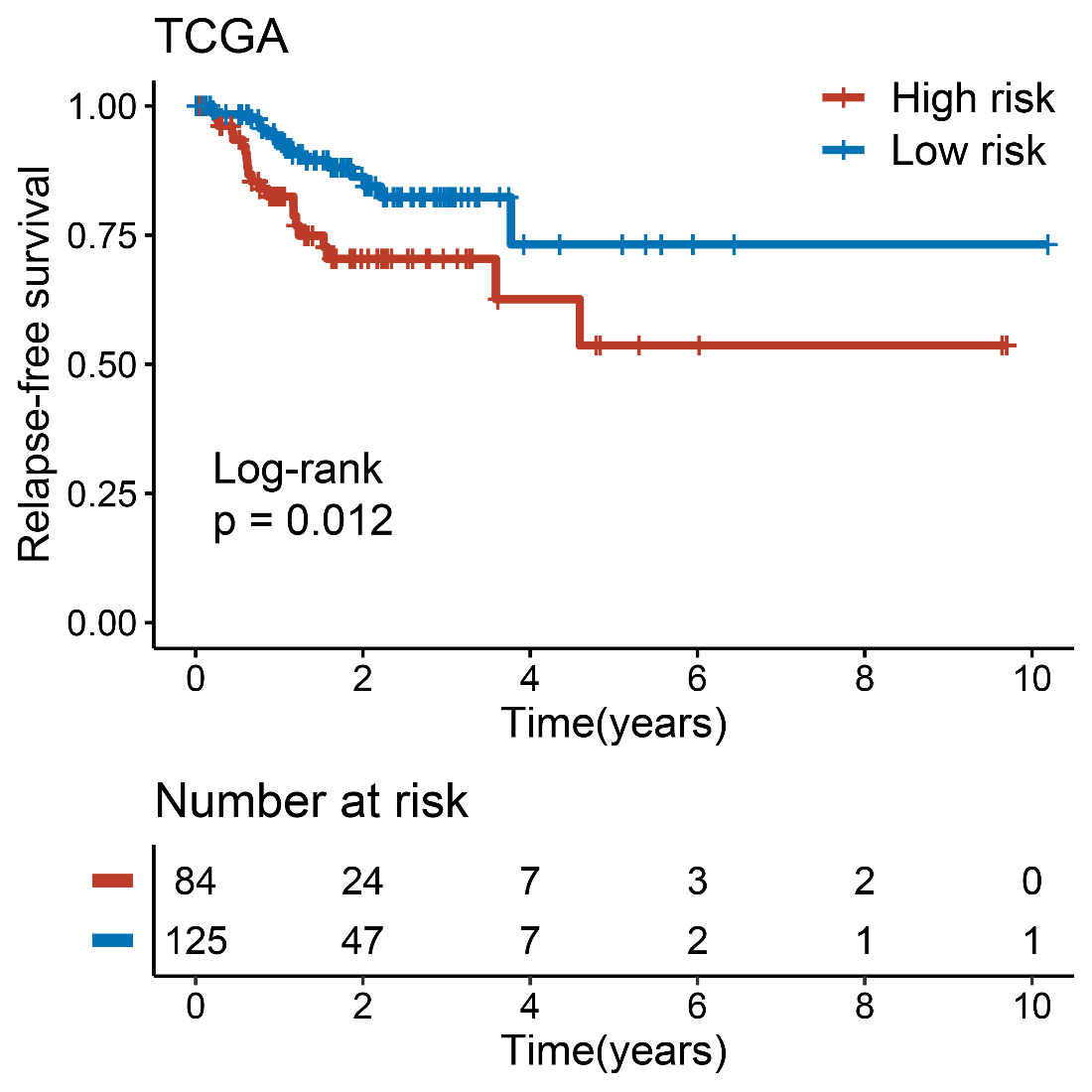


**Figure S1: Kaplan-Meier curves of RFS according to the high- and low-risk groups in TCGA.**


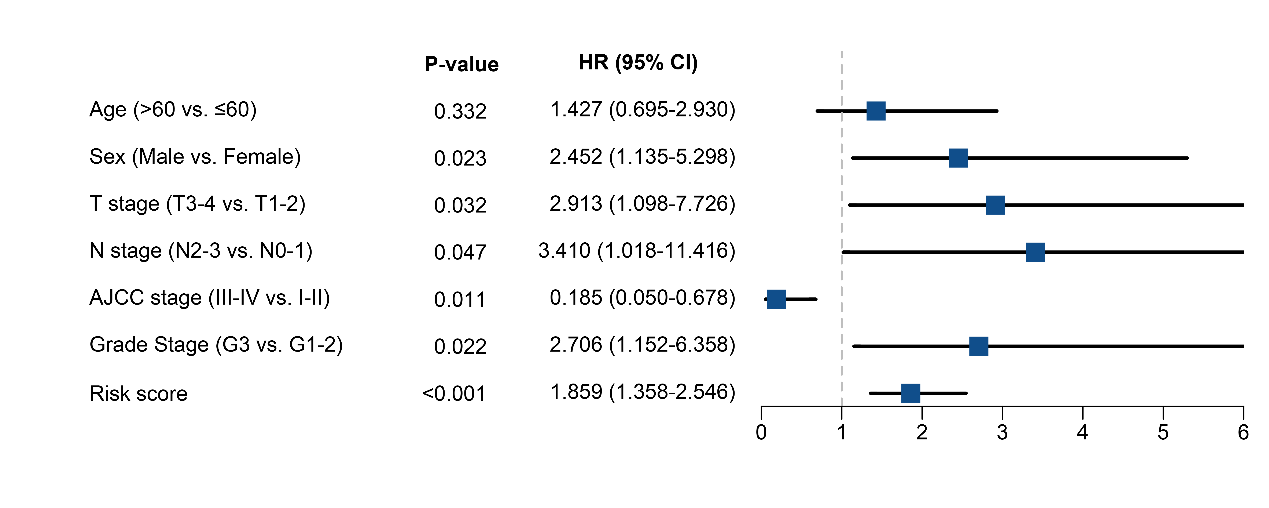


**Figure S2: Multivariate COX regression analysis of risk score in the TCGA cohort.**

**
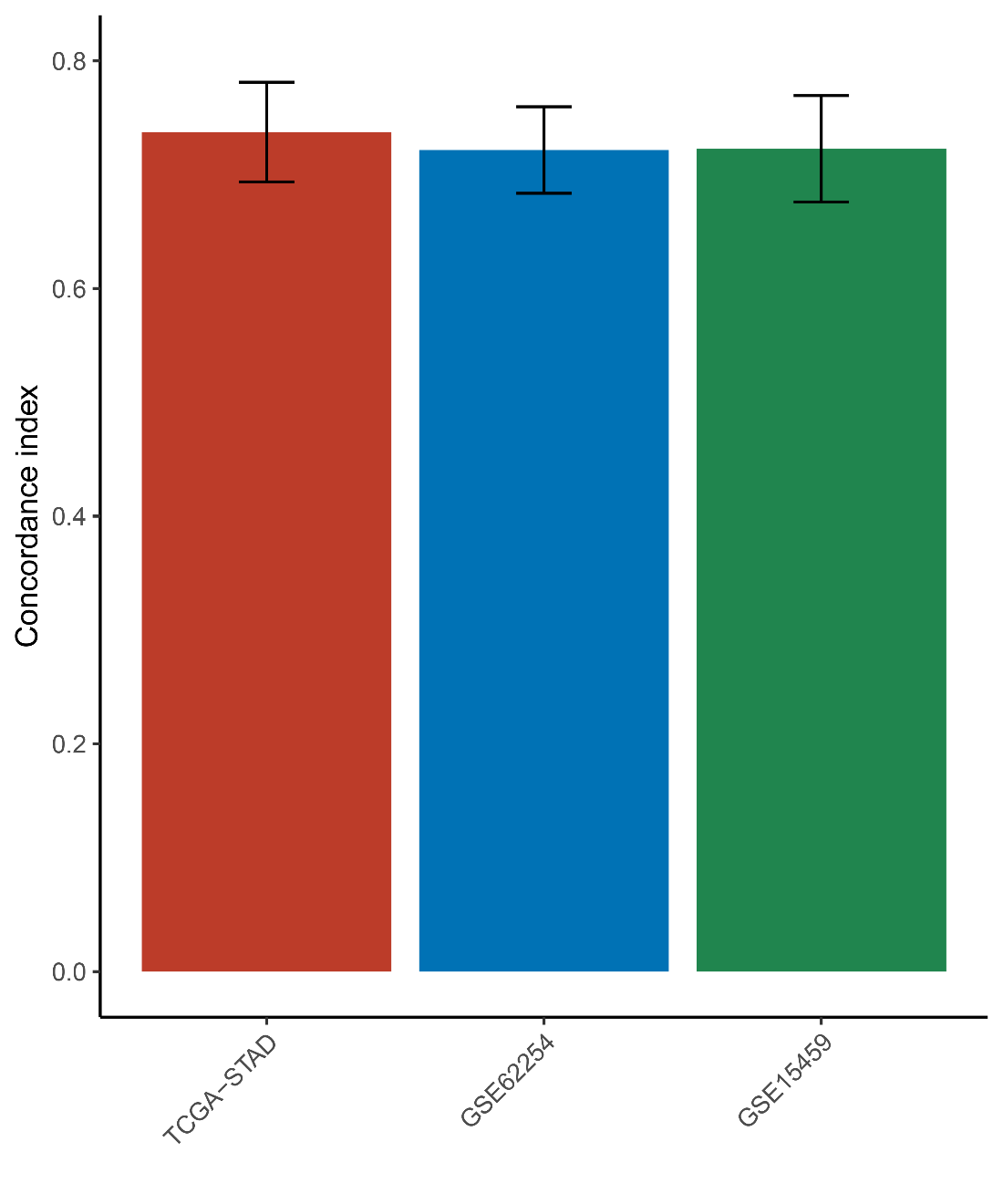
**

**Figure S3: The Harrell’s C-index of PLPPS in three cohorts.**
